# Synergistic Effect and Mechanism of Cineole and Terpineol on *In-vitro* Transdermal Delivery of Huperzine A from Microemulsions

**Published:** 2013

**Authors:** Jun Shi, Wen-Juan Cong, Yi-Ming Wang, Qing-Fei Liu, Guo-An Luo

**Affiliations:** a *School of Pharmacy, East China University of Science and Technology, Shanghai 200237, PR China.*; b *Department of Chemistry, Tsinghua University, Beijing 100084, PR China.*; c *Engineering Research Center of Modern Preparation Technology of TCM (Ministry of Education), Shanghai University of Traditional Chinese Medicine, Shanghai 201203, PR China.*; d *School of Medicine, Tsinghua University, Beijing 100084, PR China. *

**Keywords:** Permeation enhancers, Permeation mechanism, Transdermal delivery, Huperzine A, Microemulsion

## Abstract

The aim of the present study was to investigate the influence and the mechanisms of cineole and terpineol on the *in-vitro *transdermal delivery of huperzine A from microemulsions, and their potential synergistic effect on the permeation enhancement. The transdermal delivery of huperzine A from microemulsions with different concentrations of cineole and terpineol through the rat abdominal skin was determined by Franz-type diffusion cells. The partition coefficient of huperzine A between the full thickness skin and microemulsion was determined. Attenuated total reflection-Fourier transform infrared spectroscopy (ATR-FTIR) was carried out to analyze the effects of cineole and terpineol on the biophysical properties of the stratum corneum (SC) and the mechanisms of permeation enhancement. These results indicated that cineole and terpineol could synergistically increase the transdermal delivery of huperzine A from microemulsions through increasing the partition and diffusion coefficients of huperzine A. ATR-FTIR studies further validated the synergistic effect and revealed that the enhancing mechanisms were due to increasing the disorderliness and fluidity of SC lipid alkyl chains, disrupting the structure of keratin in SC, and extracting SC lipids. In conclusion, cineole and terpineol, acting synergistically to enhance the transdermal delivery of huperzine A from microemulsions, might provide an alternative permeation enhancer combination for the transdermal delivery of huperzine A.

## Introduction

Huperzine A, a natural alkaloid derived from the Chinese herb *Huperzia serrata*, has been used for improving memory, cognitive and behavioral dysfunction in patients with Alzheimer’s disease (AD) in China, and is marketed in the United States as a dietary supplement (1). It is a selective, potent, and reversible inhibitor of acetylcholine esterase (AChE) with potency comparable to donepezil, galantamine and rivastigmine - drugs commonly prescribed for the treatment of AD (2). Currently, the main marketed pharmaceutical forms of huperzine A are oral immediate-release tablets and capsules, which have to be administrated at least twice a day. However, it is rather hard for AD patients with decreased memory to persist in taking medicines (3). Moreover, huperzine A exhibits a short elimination half-life of 4.81 h in human (4), which needs to be administered frequently, resulting in variable absorption profiles and cumulative toxicosis, leading to the reduction of compliance in AD patients.

Due to the undesirable side effects and non-compliance of the oral drug administration, transdermal drug delivery system (TDDS) appears to be an attractive alternative to the oral route, being beneficial in reducing the dosage interval, maintaining the relatively stable plasma drug concentration and improving the patients’ compliance (5). During the recent years, microemulsions have received increasing attention because of the advantages such as enhanced drug solubility, good thermodynamic stability, and improved delivery properties (6-8). In these regards, the TDDS using microemulsion as a vehicle is of particular clinical significance for the long-term treatment of a chronic disease like AD.

However, the stratum corneum (SC), the outermost layer of the skin, is the principal permeability barrier and rate-limiting step for transdermal delivery of drugs across the skin. SC is composed of keratin-enriched cells embedded in a multiple lipid bilayer, which mainly consists of ceramides, cholesterol and free fatty acids (9). It is widely accepted that keratin represents the hydrophilic barrier, whereas the intercellular lipids represent the hydrophobic barrier (10). A popular technique to overcome the permeability barrier involves the use of penetration enhancers, which promotes the transdermal delivery of drugs by disturbing the organization of the multiple lipid bilayers and increasing the skin diffusivity of drugs (11). Terpenes derived from plant essential oil have been widely used in the transdermal preparations as penetration enhancers (10, 12). They are considered as less toxic compounds with low irritancy compared with the synthetic penetration enhancers, such as solvents, surfactants, azones and pyrrolidones (13). Since single enhancer usually offers limited enhancement of skin permeability, more attentions have been paid on the combination of two or more enhancers for drug delivery to overcome the limitations of individual enhancers (14).

There are very few literatures relating to screening a suitable enhancer combination for the transdermal delivery of huperzine A and investigating the potential mechanisms of the synergy between the enhancers**. **The purpose of this study was therefore to investigate the influence and the mechanisms of cineole and terpineol on the *in-vitro *transdermal delivery of huperzine A from microemulsions, and their potential synergistic effect on permeation enhancement. The transdermal delivery of huperzine A from microemulsions with different concentrations of enhancers was evaluated in Franz-type diffusion cells through the rat abdominal skin. The partition coefficient of huperzine A between the full thickness skin and microemulsion was determined, and the effects of enhancers on SC as well as the potential mechanisms of permeation enhancement were further investigated with attenuated total reflection-Fourier transform infrared spectroscopy (ATR-FTIR).

## Experimental

*Materials and animals*

Huperzine A (purity > 97.0%) was supplied by Wanbang Pharmaceutical Company (Zhejiang, China). Cremophor RH40 (RH40) was provided by BASF (Germany). Cineole (98%) and terpineol (98%) were obtained from Alfa Aesar (the United States). Trypsin was purchased from Sigma (the United States). All the other reagents were of analytical grade.

Male Sprague-Dawley rats (SPF grade), weighing 160-180 g, were supplied by the Experimental Animal Division of Peking University Health Sciences Center (Beijing, China). The studies were approved by the Institutional Animal Care and Use Committee of Tsinghua University (Beijing, China).

*Preparation of microemulsions*

Microemulsions were prepared using the water titration method (14). Briefly, 9 mg huperzine A was added to the mixtures of 1% oleic acid (as oil), 12% Cremophor RH40 (as surfactant), 4% ethanol (as cosurfactant) and different concentrations of enhancers (0.5%, 1%, 2%, and 3% of cineole or terpineol, and 0.5% cineole + 0.5% terpineol), and an appropriate amount of water was then added to the mixtures drop by drop. Various microemulsions were obtained by stirring the mixtures at ambient temperature. The microemulsions without enhancers were prepared as the control.

*Microemulsion evaluation*

The macroscopic phase behavior of vehicles was observed by polarized light microscope (Axiostar, Carl Zeiss Instruments, Germany). This polarized light screening technique was used extensively to provide information about the isotropy, anisotropy, and scattering of vehicles, through observing whether the sample rotates the plane of polarization of polarized light. The liquid-crystalline phase was easily distinguished by the birefringence displayed with polarized light.

The microstructure of the drug-loaded microemulsion was observed using freeze-fracture transmission electron microscopy (FF-TEM). Fracturing and replication were carried out in a high vacuum freeze etching system (Balzers BAF-400D, Balzers Instruments, Liechtenstein). The fracture surface was replicated by shadowing with Pt-C. The metal replicas were viewed under TEM (JEM-1200EX, JEOL Instruments, Japan).

The average droplet size and polydispersity index of the microemulsions were determined by laser scattering (Zetasizer 3000 HS, Malvern Instruments, UK).

*Preparation of full thickness skin and stratum corneum*

For *in-vitro *permeation studies, the rats were deeply anesthetized and sacrificed with an overdose of sodium pentobarbital (100 mg/Kg, IP). The abdominal skins were obtained after the hair was removed with an electric clipper. The adhering fat and connective tissue were removed carefully. The obtained full thickness skins were washed with physiologic saline, examined for integrity, and then placed in a refrigerator at - 80ºC until use.

For ATR-FTIR studies, the stratum corneum (SC) was separated from full thickness skin by digesting the skin in 10 mM phosphate-buffered saline (pH = 7.4) containing 0.1% (w/w) trypsin at 37ºC for 24 h (16). The isolated SC was rinsed with distilled water for three times, dried under a nitrogen stream, and stored in a desiccator over silica gel.

*In-vitro skin permeation studies*

The *in-vitro *penetration studies were performed in Franz-type diffusion cells (TPY-2 diffusion test apparatus, Shanghai Huanghai Drug Control Instrument Co. Ltd., Shanghai, China) with an effective diffusion area of 2.32 cm^2^ and a receptor volume of 6.5 mL. Ten mmol/L phosphate-buffered solution (PBS, pH = 7.4) was used as the receptor medium, which was magnetically stirred at 37 ± 0.5ºC with a constant rate of 600 rpm during the experiment. The microemulsions (2.0 g) were placed in the donor compartment. At predetermined times (2, 4, 6, 9 and 12 h), 200 μL of the receptor medium was withdrawn and replaced with an equal volume of freshly prepared medium. The samples were centrifuged for 15 min at 13000 rpm and an aliquot (10 μL) of the supernatant was analyzed by HPLC to determine the drug content. The experiment was replicated in triplicate.

The cumulative amount (*Q*, μg/cm^2^) of huperzine A from microemulsions permeated through the rat abdominal skin was plotted as a function of time and the slope of linear portion was estimated as steady-state permeation rate (*J*_s_, μg·cm^-2^·h^-1^).

*Chromatography*

Huperzine A was analyzed by reversed phase HPLC using Hitachi’s model L-2300 HPLC system (Tokyo, Japan), which consisted of a quaternary pump (L-2130, Hitachi, Japan), a UV detector (L-2400, Hitachi, Japan), an automatic injector (L-2200, Hitachi, Japan) and a workstation. The detection wavelength was set at 308 nm. The samples were injected directly into an Agilent TC-C18 column (4.6 mm × 250 mm, 5 μm) and eluted in a binary mixture of acetonitrile and 0.01% triethylamine solution (22:78, v/v) at a flow rate of 1.0 mL/min. The injection volume was 10 μL.

*Method validation*

The method was validated for linearity, accuracy, precision and selectivity according to the accepted guidelines of FDA (2001) for validation of bioanalytical methods (17).

The quantification of huperzine A was performed using standard calibration curve, which was generated by spiking the blank samples of skin extracts in receptor compartment with huperzine A to produce 6 concentration levels ranging between 0.2 and 200.0 μg/mL. The calibration curve was constructed by plotting the peak areas against concentration and analyzed by linear regression analysis.

Accuracy and precision evaluations were performed on both intra-day and inter-day measurements. Intra-day variability of the assay method was determined by repeated analysis of three concentrations of huperzine A (0.2, 100 and 200.0 μg/mL) in the same day. Similarly, inter-day variability was determined by repeated analysis of the same samples in three different days.

Sensitivity of the method was determined by calculating the limit of detection (LOD) and the lower limit of quantification (LLOQ). The LOD was defined as the lowest concentration of the analyte resulting in a signal-to-noise ratio of 3:1. The LLOQ was defined as the lowest drug concentration that could be determined quantitatively with appropriate precision and accuracy.

The selectivity for assessing potential interferences was tested by analyzing 6 blank samples of skin extracts in receptor compartment according to the procedure described above.

*Partition coefficient measurement*

The partition coefficient of huperzine A between the full thickness skins and microemulsions with different concentrations of enhancers (1.0% cineole, 1.0% terpineol, and 0.5% cineole + 0.5% terpineol, respectively) or without enhancers (as the control) was determined by placing each full thickness skin (100 mg) in vial containing 10 mL of microemulsion. After the vial was gently rotated at 37ºC for 24 h, an aliquot of 1.0 mL 10% (v/v) Triton X-100 in ethanol for emulsion breaking was added to 0.5 mL microemulsion, and the mixture was diluted suitably with acetonitrile-water (22:78, v/v). Thereafter, the concentration of huperzine A was assayed by HPLC. 

*ATR-FTIR studies*

To investigate the skin modification induced by the enhancers and the potential synergistic interactions between cineole and terpineol, several pieces of SC were incubated in 2 mL of different solutions (1.0% cineole, 1.0% terpineol, and 0.5% cineole + 0.5% terpineol, respectively) at 4ºC for 12 h, and 40% ethanol (v/v) was used as the solvent for these enhancers as well as the negative control. Thereafter each SC was washed carefully with distilled water and desiccated under a nitrogen stream. Fourier transform infrared spectroscopic measurements were performed using a Perkin Elmer GX FTIR spectrometer equipped with a deuterated triglycine sulfate (DTGS) detector. All spectra were obtained as an average of 16 scans recorded between 4000 cm^-1^ and 400 cm^-1^ at 30ºC, with a spectral resolution of 2 cm^-1^ and a zero filling factor of 2. The frequency precise was better than 0.1 cm^-1^.

*Statistical analysis*

The transdermal penetration of huperzine A and FTIR spectra were measured using at least three skin or SC specimens, and all the data were expressed as the mean ± standard deviations (SD). A paired Student’s t-test (two-tailed) was performed when comparing two different conditions. In all cases, p < 0.05 was considered as significant.

## Results and Discussion

*Microemulsion evaluation*

**Figure 1:**
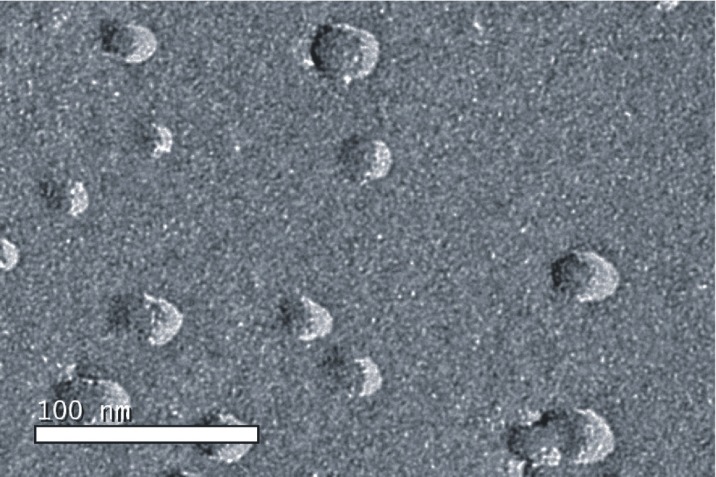
FF-TEM image of drug-loaded microemulsion

Microstructure of the drug-loaded microemulsion was characterized using FF-TEM (Figure 1). The average size of all microemulsions ranged from 14.3 ± 1.2 to 73.1 ± 5.9 nm, and the polydispersity index, ranging from 0.153 ± 0.002 to 0.186 ± 0.003, showed that all microemulsions had narrow size distribution.

*Method validation*

No interference of any peak was observed at the retention time for huperzine A (9.1 min), demonstrating a good selectivity of the assay method. The calibration plot was linear for huperzine A over the concentration range of 0.2-200.0 μg/mL, with the correlation coefficient of the standard curve being 0.999. The LOD and LLOQ of the method were 0.1 μg/mL and 0.2 μg/mL with the corresponding relative standard deviation (RSD) of 13.8% and 10.6%, respectively. The intra-day and inter-day accuracy and precision were lower than 10% RSD in all the concentrations analyzed, which satisfactorily met the international established acceptance criteria (17).

*In-vitro skin permeation studies*

**Figure 2:**
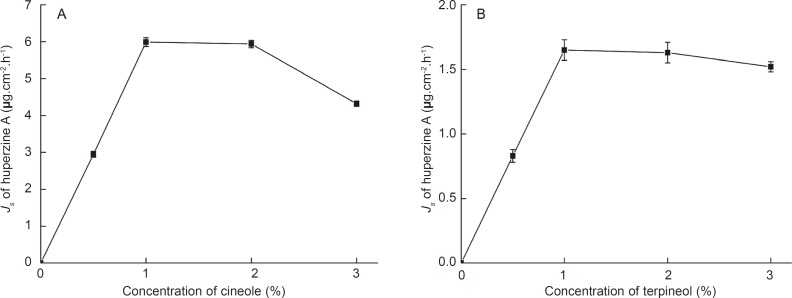
Effects of concentrations of cineole (A) and terpineol (B) on the permeation rates of huperzine A from microemulsions. Data are represented as mean ± SD (n = 3).

Different concentrations of cineole and terpineol as enhancers were added to the microemulsions, respectively, to improve the permeation rate of huperzine A. The plot of the permeation rate of huperzine A against the concentration of enhancers is exhibited in Figure 2. 

The permeation rate of huperzine A increased linearly with the concentration of the enhancers from 0% to 1%. Furthermore, when the amount of huperzine A in the microemulsions was kept constant and the concentration of the enhancers was varied, the maximal permeation rate was observed already at 1% cineole or 1% terpineol present in the microemulsions. Compared with the control microemulsions, the permeation rates of huperzine A from microemulsions with 1% cineole and 1% terpineol were significantly increased, being 4.58 and 16.64 times higher, respectively. The decreased permeation rate of huperzine A with the increasing concentration of cineole or terpineol might be attributed to the permeation obstruction caused by the higher viscosity of microemulsions with higher concentration of enhancer.

**Figure 3:**
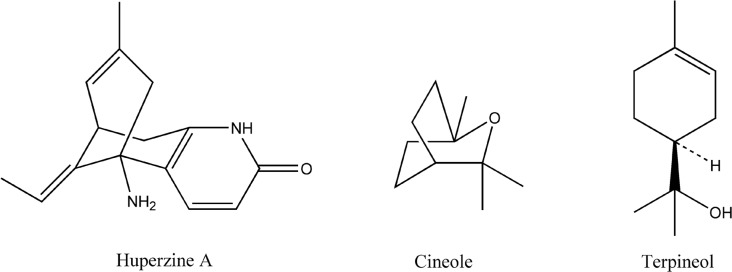
Chemical structures of huperzine A, cineole and terpineol

 One of the predominant factors known to determine the permeation of drugs across skin is the partition coefficient (18). The huperzine A amounts partitioned between full thickness skins and microemulsions with 1% cineole and 1% terpineol were 7.29 ± 0.51 μg/g and 14.66 ± 1.18 μg/g, respectively, being significant higher than the huperzine A amount partitioned between thickness skin and the control microemulsion (3.16 ± 0.12 μg/g). Considering the structures (Figure 3), both of cineole and terpineol belong to oxygen-containing terpenes (11), which makes them easier to form hydrogen bond with ceramide head groups, and loose or even disrupt the existing hydrogen bond network at the head of ceramides in the SC lipid bilayers, thereby facilitating the permeation of huperzine A. The huperzine A amount partitioned between full thickness skin and microemulsion with 1% terpineol was much higher than that partitioned between full thickness skin and microemulsion with 1% cineole, whereas 1% cineole was more efficient in enhancing the permeability of huperzine A from microemulsion than 1% terpineol, which suggested that other mechanisms were probably responsible for the lower enhancement effect of terpineol compared with cineole.

Another predominant factor related to the permeation of drugs across skin is the diffusion coefficient. Diffusion is a size-dependent process and an increase in the diffusant volume as a result of molecular association, such as hydrogen bonding, will reduce diffusion coefficient (19). Huperzine A can accept and donate hydrogen bonds at more than one site (Figure 3). Terpineol, with the hydroxyl functionalities, can also accept or donate hydrogen bonds, whereas cineole, with the epoxy functionalities, can only accept hydrogen bonds (20). Therefore, the lower enhancement effect of terpineol in comparison with that of cineole could be partly attributed to the larger size of huperzine A-terpineol complex than that of huperzine A-cineole complex, possibly due to the differences in the stoichiometry of the interactions.

Additionally, the physicochemical characteristics such as log P and boiling point of terpene also play an important role in enhancing the permeabilities of several drugs (21). Since the log p-values of cineole (log P 2.82) and terpineol (log P 2.69) do not significantly differ from each other, the permeation enhancement cannot be attributed to their lipophilicity (21). On the other hand, the boiling point of cineole (173ºC) is approximately 44 ºC less than that of terpineol (217ºC), indicating that the cohesiveness or self-association of cineole is weaker than terpineol (21), which can be one of the reasons for cineole being more efficient in enhancing the permeability of huperzine A from microemulsions than terpineol.

**Figure 4:**
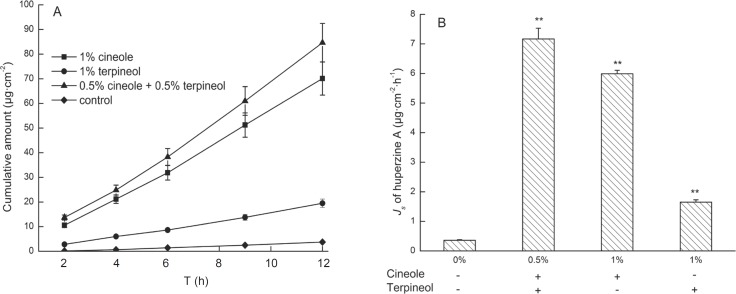
Effects of the treatment with 1% cineole, 1% terpineol and 0.5% cineole + 0.5% terpineol on the transdermal delivery of huperzine A from microemulsions. Data are represented as mean ± SD (n = 3). (A) The permeation profiles of huperzine A through rat skins (B) The permeation rates of huperzine A through rat skins. ^a^ p < 0.001 compared with control; ^b^ p < 0.05 compared with 0.5% cineole + 0.5% terpineol

Furthermore, the effects of 0.5% cineole + 0.5% terpineol on the transdermal delivery of huperzine A from microemulsions were investigated. A steady increase of huperzine A in the receptor compartment with time was observed and the permeation profiles followed zero-order release kinetics (Figure4A). Moreover, the permeation rate of huperzine A from microemulsion with 0.5% cineole + 0.5% terpineol was 20.48 times higher than that of the control microemulsion, and the enhancement of the permeation rate was significantly greater than the increase caused by 1% individual enhancers (Figure 4B). These results suggested that there were potential synergistic interactions between 0.5% cineole and 0.5% terpineol in transdermal delivery of huperzine A. In addition, the huperzine A amount partitioned between full thickness skin and microemulsion with 0.5% cineole + 0.5% terpineol was 16.80 μg/g, being significant higher than that partitioned between thickness skin and microemulsion with 1% cineole (7.29 μg/g) or 1% terpineol (14.66 μg/g), which suggested that 0.5% cineole + 0.5% terpineol could influence the partition coefficient with synergic effect. Taking these aspects together, 0.5% cineole and 0.5% terpineol might compensate each other with different mechanisms, such as influencing the partition and diffusion coefficients of the drug, in enhancing the permeability of huperzine A from microemulsions. Therefore, 0.5% cineole + 0.5% terpineol as the enhancer combination might be suitable for future studies due to its higher permeation enhancing effect.

*ATR-FTIR studies*

**Figure 5:**
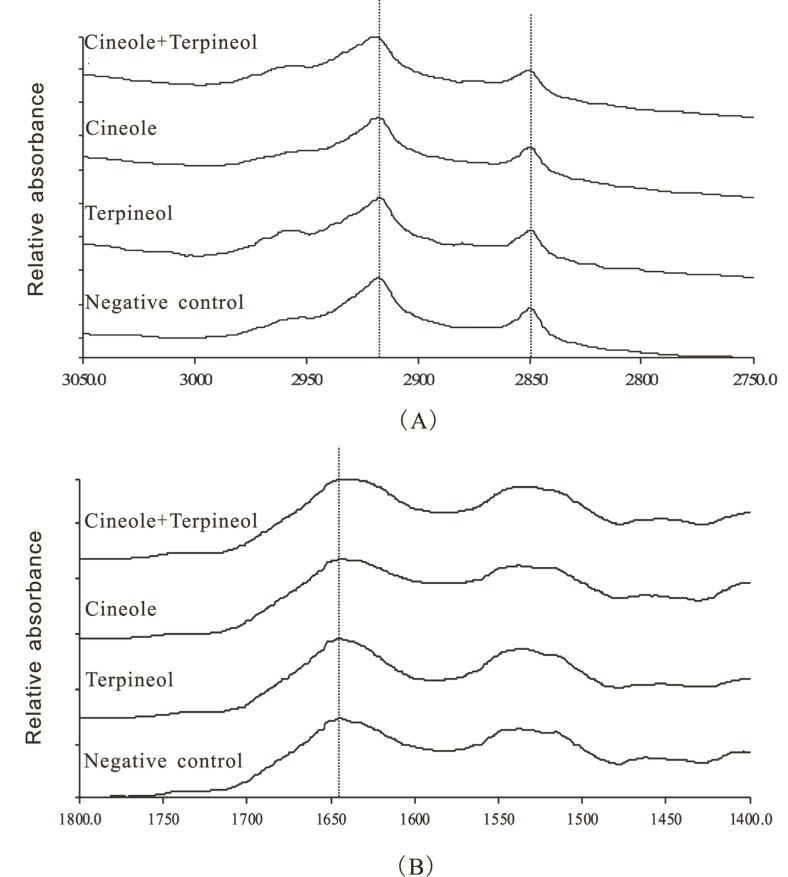
ATR-FTIR spectra of rat SC treated with 0.5% cineole + 0.5% terpineol, 1% cineole, 1% terpineol, and 40% ethanol, respectively. (A) ATR-FTIR spectra representing νa (CH_2_) and νs (CH_2_); (B) ATR-FTIR spectra representing ν (CO).

ATR-FTIR as a powerful tool for determining the fluidity of SC and the structural alteration of lipids and keratin in SC has been extensively used to investigate the effects of penetration enhancers on SC (16, 23). The ATR-FTIR spectra of rat SC treated with various enhancers are displayed in Figure 5. Characteristic peaks could be found in CH_2_ asymmetric stretching vibration [ν_a_ (CH_2_)] frequency peak near 2920 cm^-1^, CH_2_ symmetric stretching vibration [ν_s_ (CH_2_) ] frequency peak near 2850 cm^-1^, and C=O stretching vibration [ν (CO) ] frequency peak near 1650 cm^-1^ (16). The CH_2_ stretching vibration frequencies were derived from methylene group on the hydrophobic alkyl chains of intercellular lipids (ceramides, cholesterol and fatty acids) in SC, being significantly influenced by the incorporation of enhancers (23). The C=O stretching vibration frequency was derived from carbonyl group from the amide ׀ of the keratin in SC (16).

As showed in Figure 5 and Table 1, SC exposed to 40% ethanol in the absence of enhancers (the negative control) exhibited ν_a_ (CH_2_), νs (CH_2_), and ν (CO) peaks at 2917.1, 2849.3, and 1644.0 cm^-1^, respectively. SC treated with 1% cineole alone and 1% terpineol alone caused blue shifts in ν_a_ (CH_2_) of 1.2 cm^-1^ and 0.9 cm^-1^ relative to the negative control, respectively. Interestingly, SC treated with 0.5% cineole + 0.5% terpineol caused a blue shift in ν_a_ (CH_2_) of 2.1 cm^-1^ relative to the negative control, which was significantly different from SC treated with 1% individual enhancers. 

**Table 1:** Effect of various enhancers on the ν_a_ (CH_2_), ν_s_ (CH_2_) and ν (CO) stretching vibration shifts (cm^-1^).

**Treatment**	**ν**_a_ ** (CH**_2_ **)**	**ν**_s _ **(CH**_2_ **)**	**ν(CO)**
1% Cineole	2918.3 ± 0.2 ^a, b^	2849.9 ± 0.2 ^a^	1643.4 ± 0.3 ^a, b^
1% Terpineol	2918.0 ± 0.1 ^a, b^	2849.6 ± 0.2 ^a, b^	1643.8 ± 0.1^a, b^
0.5% Cineole + 0.5% Terpineol	2919.2 ± 0.1 ^a^	2850.4 ± 0.2 ^a^	1640.9 ± 0.2 ^a^
Negative control	2917.1 ± 0.2 ^b^	2849.3 ± 0.2 ^b^	1644.0 ± 0.1 ^b^

Data are listed as mean ± SD (n = 4). a: p < 0.05 compared with negative control; b: p < 0.05 compared with 0.5% cineole + 0.5%

Furthermore, SC treated with 1% cineole, 1% terpineol, and 0.5% cineole + 0.5% terpineol also produced blue shifts in ν_s _(CH_2_) peak position, and the peak shifts by these enhancers were 0.6 cm^-1^, 0.3 cm^-1^ and 1.1 cm^-1^ relative to the negative control, respectively. Altogether, the significant blue shifts in both ν_a_ (CH_2_) and ν_s_ (CH_2_) of lipid alkyl chains in SC indicated that both of 1% cineole and 1% terpineol could increase the disorderliness and fluidity of SC lipid alkyl chains to some extent, and this disorder could be further increased when SC was treated with 0.5% cineole + 0.5% terpineol.

Additionally, SC treated with 1% cineole alone and 1% terpineol alone caused decreases in ν (CO) of 0.6 cm^-1^ and 0.2 cm^-1^ relative to the negative control, respectively, which suggested that the keratin in SC was also affected by 1% cineole and 1% terpineol. Furthermore, SC treated with 0.5% cineole + 0.5% terpineol caused a significant decrease in ν (CO) of 3.1 cm^-1^ relative to the negative control, and the decrease was significantly different from SC treated with 1% individual enhancers. The abovementioned data indicated that both 1% cineole and 1% terpineol could change the structure of keratin in SC through promoting a transformation from *α*-helix to random coiling structure of the keratin and loosing the accumulative structure of keratin, eventually enhancing the transdermal permeabilities of drugs (25). Most importantly, 0.5% cineole and 0.5% terpineol could act synergistically to transform the structure of keratin.

Furthermore, ATR-FTIR results in Table 2 showed that cineole and terpineol could also cause significant lipid extraction to different extents indicated by the remarkable decreases in the peak areas of ν_a_ (CH_2_) (the baseline was set at 2998.0-2860.0 cm^-1^) and ν_s_ (CH_2_) (the baseline was set at 2860.0-2840.0 cm^-1^) by 69.92% and 68.74%, 17.71% and 15.42%, 14.83% and 19.67% corresponding to 0.5% terpineol + 0.5% cineole, 1% cineole, and 1% terpineol compared with the negative control, respectively. The decreasing trend in peak areas was 0.5% terpineol + 0.5% cineole > 1% cineole > 1% terpineol. The peak areas were most significantly diminished when 0.5% cineole + 0.5% terpineol was adopted, indicating that there were synergistic interactions between cineole and terpineol to extract SC lipids.

**Table 2:** Effect of various enhancers on the ν_a_ (CH_2_), ν_s _(CH_2_) and ν (CO) peak areas

**Treatment**	**ν**_a _ **(CH**_2_ **)**	**ν**_s _ **(CH**_2_ **)**	**ν(CO)**
1% Cineole	10.45 ± 0.47 ^a,b^	1.43 ± 0.08 ^a, b^	16.88 ± 0.83 ^a, b^
1% Terpineol	10.82 ± 0.52 ^a, b^	1.36 ± 0.06 ^a, b^	20.82 ± 1.56 ^a, b^
0.5% Cineole + 0.5% Terpineol	3.82 ± 0.12 ^a^	0.53 ± 0.03 ^a^	8.36 ± 0.38^ a^
Negative control	12.70 ± 0.66 ^b^	1.70 ± 0.09 ^b^	21.63 ± 1.62 ^b^

Data are listed as mean ± SD (n = 4). a: p < 0.05 compared with negative control; b: p < 0.05 compared with 0.5% cineole + 0.5%

These results above could explain that both of cineole and terpineol possessed enhanced effects on the *in-vitro *transdermal delivery of huperzine A from microemulsions, and further proved that 0.5% cineole and 0.5% terpineol could act synergistically to improve the permeability of huperzine A.

## Conclusion

The effects and enhanced mechanisms of cineole and terpineol as terpene enhancers on the *in-vitro *transdermal delivery of huperzine A from microemulsions were investigated. The results indicated that both cineole and terpineol could increase the transdermal delivery of huperzine A from microemulsions, and 0.5% cineole + 0.5% terpineol could influence the partition and diffusion coefficients of the drug through potential synergistic interactions between the two enhancers resulting in dramatically increasing the permeability of huperzine A. The ATR-FTIR spectroscopy investigation further authenticated the synergistic effect and revealed that the enhancing mechanisms were related to increasing the disorderliness and fluidity of SC lipid alkyl chains, disrupting the structure of keratin in SC, and extracting SC lipids. Generally speaking, 0.5% cineole + 0.5% terpineol might provide an alternative permeation enhancer combination for the transdermal delivery of huperzine A.
